# The regulation of plasma gelsolin by DNA methylation in ovarian cancer chemo-resistance

**DOI:** 10.1186/s13048-023-01332-w

**Published:** 2024-01-12

**Authors:** Hafiza Bushra Manzoor, Meshach Asare-Werehene, Satyajit Dey Pereira, Kapaettu Satyamoorthy, Benjamin K. Tsang

**Affiliations:** 1 Chronic Disease Program, Ottawa Hospital Research Institute, Ottawa, ON K1H 8L6 Canada; 2 Department of Cellular and Molecular Medicine, Faculty of Medicine, University of Ottawa, Ottawa, ON K1H 8M5 Canada; 3 Department of Obstetrics & Gynecology, & The Centre for Infection, Immunity and Inflammation (CI3), Faculty of Medicine & Interdisciplinary School of Health Sciences, Faculty of Health Sciences, University of Ottawa, Ottawa, ON K1H 8L1 Canada; 4 Department of Cell and Molecular Biology, Manipal School of Life Sciences, Manipal Academy of Higher Education, Manipal, Karnataka 576104 India; 5 Department of Laboratory Medicine and Pathobiology, University of Toronto, Toronto, ON Canada; 6 Shri Dharmasthala Manjunatheshwara University, Manjushree Block, Manjushree Nagar Sattur, Dharwad, Karnataka 580 009 India

**Keywords:** Ovarian cancer, Chemoresistance, DNA methylation, Plasma gelsolin (pGSN), Ten eleven translocation enzyme isoform 1 (TET1)

## Abstract

**Background:**

Ovarian cancer (OVCA) is the most lethal gynecologic cancer and chemoresistance remains a major hurdle to successful therapy and survival of OVCA patients. Plasma gelsolin (pGSN) is highly expressed in chemoresistant OVCA compared with their chemosensitive counterparts, although the mechanism underlying the differential expression is not known. Also, its overexpression significantly correlates with shortened survival of OVCA patients. In this study, we investigated the methylation role of Ten eleven translocation isoform-1 (TET1) in the regulation of differential pGSN expression and chemosensitivity in OVCA cells.

**Methods:**

Chemosensitive and resistant OVCA cell lines of different histological subtypes were used in this study to measure pGSN and TET1 mRNA abundance (qPCR) as well as protein contents (Western blotting). To investigate the role of DNA methylation specifically in pGSN regulation and pGSN-induced chemoresistance, DNMTs and TETs were pharmacologically inhibited in sensitive and resistant OVCA cells using specific inhibitors. DNA methylation was quantified using EpiTYPER MassARRAY system. Gain-and-loss-of-function assays were used to investigate the relationship between TET1 and pGSN in OVCA chemoresponsiveness.

**Results:**

We observed differential protein and mRNA expressions of pGSN and TET1 between sensitive and resistant OVCA cells and cisplatin reduced their expression in sensitive but not in resistant cells. We observed hypomethylation at pGSN promoter upstream region in resistant cells compared to sensitive cells. Pharmacological inhibition of DNMTs increased pGSN protein levels in sensitive OVCA cells and decreased their responsiveness to cisplatin, however we did not observe any difference in methylation level at pGSN promoter region. TETs inhibition resulted in hypermethylation at multiple CpG sites and decreased pGSN protein level in resistant OVCA cells which was also associated with enhanced response to cisplatin, findings that suggested the methylation role of TETs in the regulation of pGSN expression in OVCA cells. Further, we found that TET1 is inversely related to pGSN but positively related to chemoresponsiveness of OVCA cells.

**Conclusion:**

Our findings broaden our knowledge about the epigenetic regulation of pGSN in OVCA chemoresistance and reveal a novel potential target to re-sensitize resistant OVCA cells. This may provide a future therapeutic strategy to improve the overall OVCA patient survival.

## Introduction

Ovarian cancer (OVCA) is the third most common gynaecological cancer after cervical and uterine cancer [1]. Despite the advances in diagnosis and treatment approaches, the 5-year survival rate for OVCA patients remains between 30 and 40% (stage III) and 20% (stage IV) [2]. The first line of treatment for OVCA is the combination of surgical debulking and platinum-based chemotherapy [3]. Most of ovarian cancer patients show significant clinical response to first-line chemotherapy, however, 25% of early stage and 80% of advanced stage ovarian cancer patients relapse with chemoresistance, causing over 90% of deaths [4, 5]. The mechanisms of platinum resistance are multifactorial and may involve any of the following: alteration of multiple molecular pathways including mutation and silencing of tumor suppressor genes, activation of oncogenes, epigenetic modifications, dysregulation of cell survival pathways (PI3K/Akt) and anti-apoptotic signalling pathways (Bcl-2, Bcl-xl, p53), epithelial to mesenchymal transition (EMT), dysregulation in drug uptake and efflux, tumor microenvironment, increased DNA damage repair and the upregulation of gelsolin expression and function [6–13]. It is therefore urgent to investigate the cellular and molecular mechanisms underpinning chemoresistance in OVCA.

**Fig. 1:**
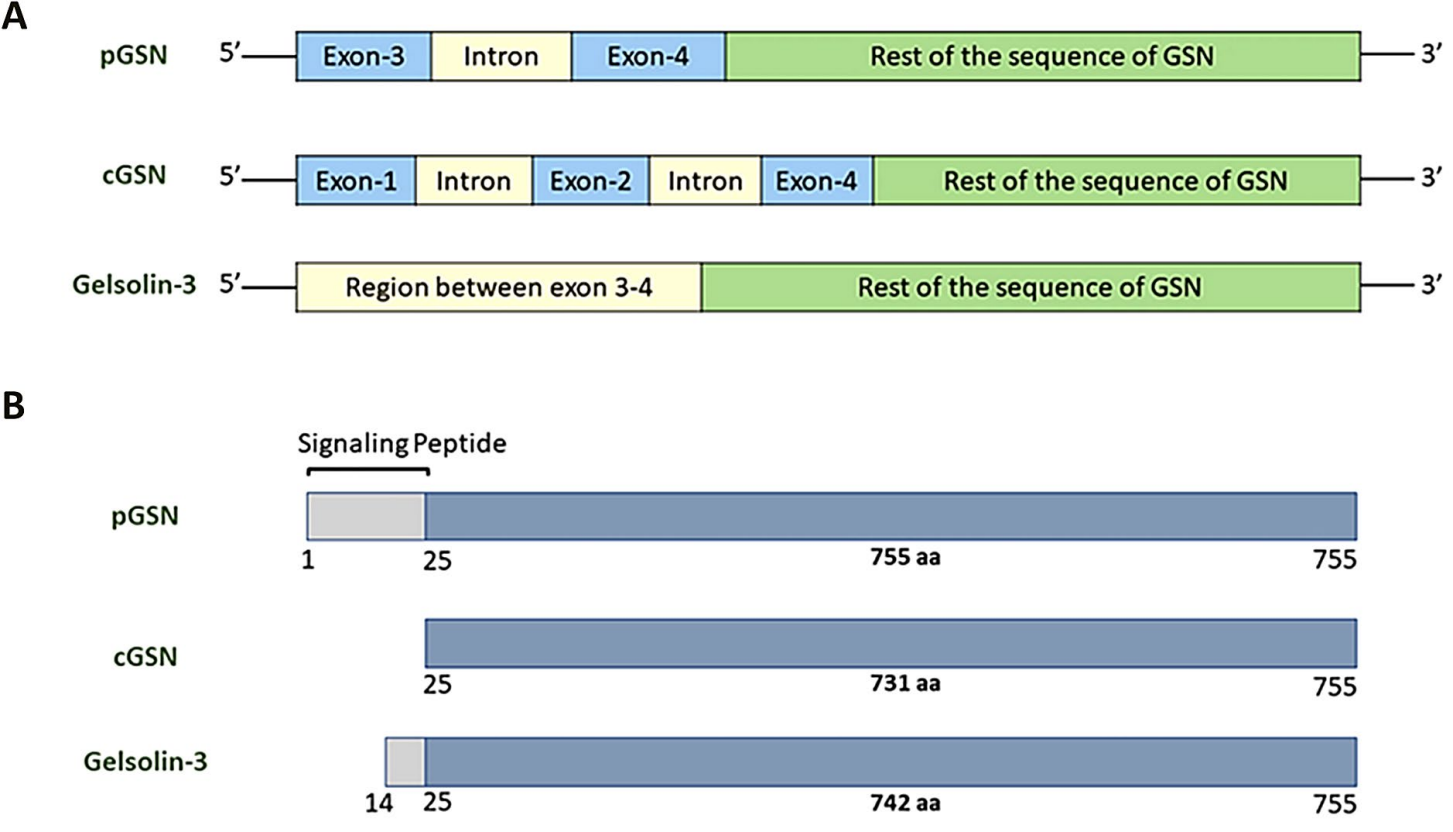
Genomic context and structure of GSN isoforms. (**A**) The pGSN, cGSN, and Gelsolin-3 are three well known isoforms of GSN, which are encoded by single gene and formed by alternative splicing and different transcriptional sites. Three isoforms are characterized by arrangement of 5`-end; pGSN 5`-end is made up of exon 3-intron-exon 4 while exon 3 make up untranslated region (UTR) and codes for signaling peptide, cGSN 5`-end made up of exon 1- intron- exon 2 – intron – exon 4, while exons 1 and 2 make UTR, 5`-end of Gelsolin-3 is made up of region between exon 3 and 4 which also make its UTR. (**B**) All three isoforms have similar 733 amino acids, pGSN and Gelsolin-3 have 24 and 11 extra amino acids at N-terminus, respectively, which differentiate them from cGSN

Gelsolin (GSN), a calcium-dependent actin-binding protein, is best known as a regulator of actin skeleton and primarily responsible for cellular architecture and motility [14]. So far, three isoforms of GSN have been well characterized: cytoplasmic GSN (cGSN), secreted/plasma GSN (pGSN), and gelsolin-3. These are produced by alternative splicing and different transcriptional initiation sites on the GSN gene [15] (Fig. 1). cGSN and pGSN are two well defined isoforms involved in carcinogenesis and differs from one another by the presence of 24-amino acid signalling peptide extension at the N-terminal region and a disulfide bond between Cys188 and Cys201 residues in pGSN [15]. pGSN plays an important role of an extracellular actin scavenger in preventing actin toxicity and has also been implicated in various inflammatory diseases, bacterial and viral infections, malignancies and injuries [16].

Recently, pGSN has gained much attention because of its role in OVCA chemoresistance as well as in other malignancies [13, 17–19]. pGSN is highly expressed in chemoresistant OVCA cells, secreted via exosomes and activates the α5β1 integrins / FAK (focal adhesion kinase) / Akt / HIF-1α (hypoxia-inducible factor-1) axis resulting in increased pGSN production which confers resistance to otherwise sensitive OVCA cells in a paracrine manner. Also, increased pGSN expression protects OVCA cells against cisplatin-induced apoptosis [13]. Chemoresistant patients express higher tumor levels of pGSN compared with their sensitive counterparts leading to poor overall survival. Additionally, pGSN activates the NRF2 pathway in ovarian cancer cells resulting in increased production of GSH and other anti-oxidant factors, a process that deactivates cisplatin and inhibit cell death [20]. pGSN, within the tumor, inhibits the survival impacts of infiltrated CD8 + T cells, dendritic cells and macrophages, resulting in shortened patient survival as well as poor treatment responsiveness [20]. Secretory pGSN has also proven to be a potential marker for predicting early-stage disease, residual disease, and treatment responses in OVCA patients. Exosomal pGSN outperforms CA125 in the prediction of chemoresistance in OVCA patients, suggesting the important role of pGSN in OVCA early diagnosis, prognosis and chemoresistance. [21, 22]. Although the diagnostic and therapeutic properties of pGSN have been investigated, we have yet to determine the regulatory mechanisms behind the differential levels of pGSN between chemoresistant and chemosensitive patients.

DNA methylation is a well-known epigenetic mechanism associated with gene regulation in cancer development, progression, recurrence and chemoresistance. DNA methyltransferase family enzymes (DNMTs) introduces methyl group at carbon 5 of cytosine residue, which is usually followed by guanine in CpG dinucleotides, and convert it to 5-methylcytosine (5mC) [23]. Whereas, Ten-eleven translocation methylcytosine dioxygenase family (TETs) that are α-ketoglutarate (α-KG)/Fe (II) dioxygenases catalyze the DNA demethylation by causing hydroxylation of 5mC to generate 5-hydroxymethylcytosine (5hmC) which is further oxidized to 5-formylcytosine (5fC) and 5-carboxycytosine (5caC), and restored back to unmethylated cytosine by base excision repair mechanisms [24–26]. Downregulation of tumor suppressor genes by aberrant methylation in their promoter region and global hypomethylation/specific hypomethylation of oncogenes are two most common epigenetic phenomena occurring in all cancers, including ovarian cancer [27, 28]. The role of altered DNA methylation, both at specific gene and the global level, in the ovarian cancer chemoresistance development has extensively been explored [29–31]. TET1 is the most studied isoform of TETs family and primarily responsible for 5mC to 5hmC oxidation [32]. Recently, several biological functions regulated by TET1 have been identified in different malignancies. TET1 has been reported to have dual tumor-promoting and tumor-suppressing functions in cancer development, progression and treatment responses [32–34]. Also, a number of studies have highlighted TET1’s role as a promising target to overcome chemoresistance in different cancers [35–39]. As to whether TET1 has a role to play in OVCA chemoresistance remains to be determined.

To date, no major naturally occurring mutation, deletion, or rearrangement in the gelsolin gene has been identified that regulate its role in the context of cancer. However, a number of studies have reported epigenetic regulation of gelsolin expression including DNA methylation, histone and miRNA modification [40–44]. Hence, new studies on epigenetic regulation of pGSN could provide a better understanding of the control of its expression and function in OVCA chemoresistance. In the present study, we investigated the role of DNA methylation specifically TET1 in the regulation of pGSN expression in OVCA cells and their response to cisplatin.

## Results

### TET1 expression is associated with poor prognosis of Ovarian cancer patients

**Fig. 2:**
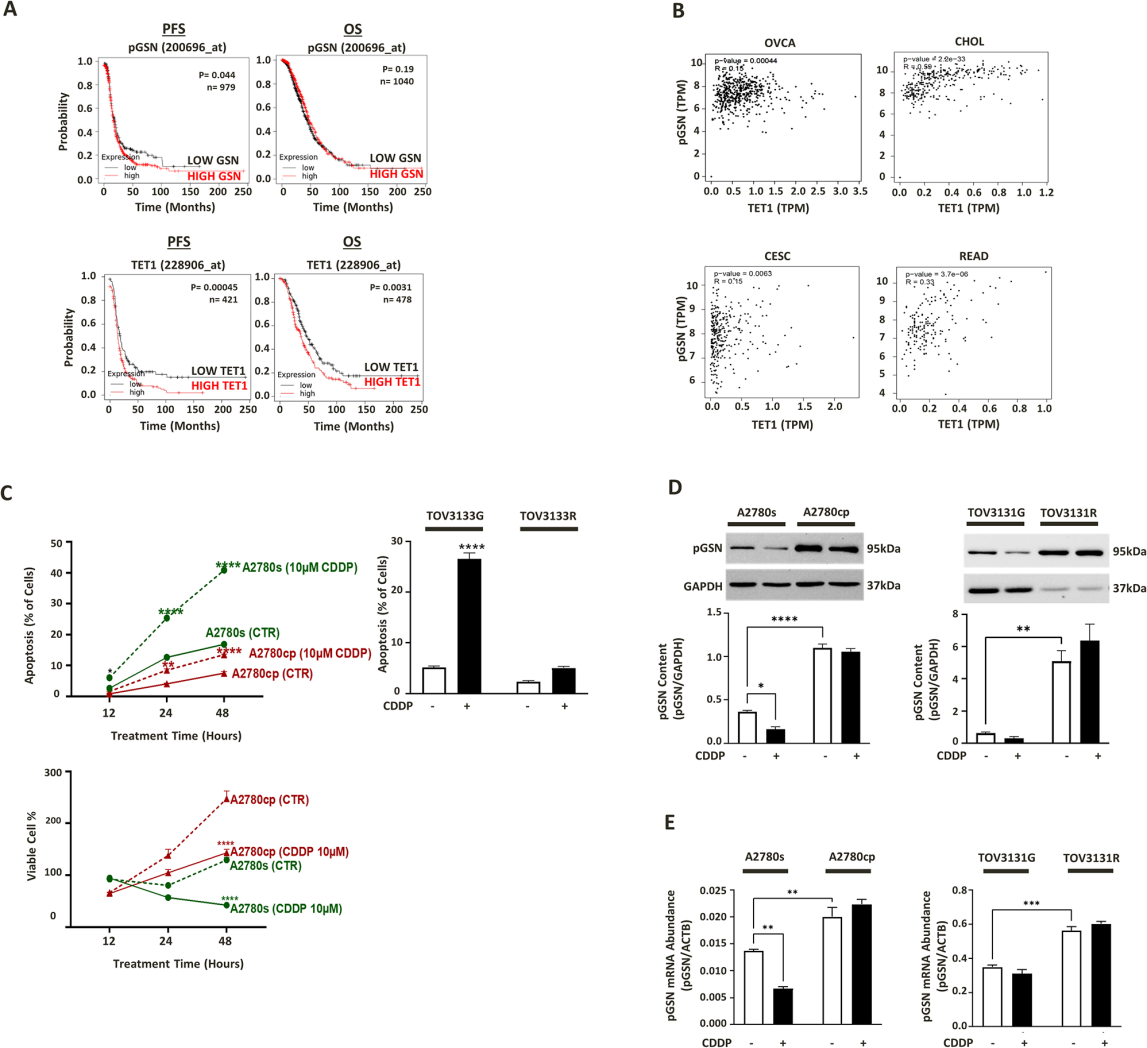
High TET1 and GSN expression significantly correlate with increased recurrence and shortened survival of OVCA patients. (**A**) OVCA public data sets (Kaplan-Meier Plotter) was analyzed for TET1 correlation with patient survival and (**B**) GEPIA was used to demonstrate the correlation between GSN and TET1 gene expression in ovarian cancer (OVCA), cholangiocarcinoma (CHOL), cervical squamous cell carcinoma and endocervical adenocarcinoma (CESC), and rectum adenocarcinoma (READ) tissues. Significantly higher pGSN mRNA and protein contents observed in OVCA resistant cells (endometrioid, A2780cp; HGS, TOV3133R) compared to sensitive cells (endometrioid, A2780s; HGS, TOV3133G) which is associated with decreased chemoresponsiveness. OVCA cells were treated with or without CDDP (10 µM; 24 h). (**C**) Apoptosis was morphologically determined by Hoechst 33,258 nuclear staining and cell viability examined using CCK8 assay (**D**) pGSN and GAPDH (loading control) protein contents were assessed by Western blotting (**E**) pGSN mRNA content relative to ACTB (loading control) was assessed by qPCR. (mean ± SEM; n = 3). Differences between all the groups were evaluated using two-way and three-way ANOVA followed by Tukey’s multiple comparison test; p*<0.05, p**<0.01, p***<0.001, p****<0.0001

Publicly available ovarian cancer datasets (www.kmplot.com) were interrogated to identify the association of pGSN (affymetrix Id: 200696_at) expression, and TET1 expression (affymetrix Id: 228904_at) with OVCA patients’ survival. Patient information was stratified based on histological subtype (serous), surgical outcome (optimal/suboptimal debulking), and chemotherapy treatment (platinum). Further, all tumor stages, grades and p53 statuses were included in the analyses. Progression-free survival (PFS) and overall survivals (OS) of OVCA patients were correlated with TET1 expression using Kaplan Meier plotter. The log-rank test was used for statistical parameter calculation and graph plot was used for visualization. In serous carcinoma patients who received platinum-based chemotherapy treatment, high pGSN expression was significantly (p = 0.044) associated with shortened PFS (16.6 months) compared with patients with lower pGSN expression (18.27 months). High pGSN expression was also associated with low OS (43.93 months) compared with patients with lower pGSN expression (46.6 months), however the association was not significant (p = 0.19). While PFS is indicative of time from initial treatment to recurrence and directly reflects tumor biology, OS might be impacted by other comorbidities [45]. pGSN has a biological effect on tumor recurrence and associated with chemoresistance [13, 46], this could explain significant association of pGSN with PFS but not with OS where other health conditions associated with the patients might contribute to their survival rate. Similarly, we observed that high TET1 expression was significantly (p = 0.00045) associated with shortened PFS (14 months) compared with patients with lower TET1 expression (18 months) in serous carcinoma patients. High TET1 expression was also significantly (p = 0.0031) associated with low OS (37.9 months) compared with patients with lower TET1 expression (48 months) (Fig. 2A). We also used GEPIA (www.gepia.cancer-pku.cn) public dataset to determine if there is any correlation between pGSN and TET1 gene expression in multiple human cancers including ovarian cancer, cholangiocarcinoma (CHOL), cervical squamous cell carcinoma and endocervical adenocarcinoma (CESC), and rectum adenocarcinoma (READ) tissues. Similarly, statistical parameters were calculated using spearman correlation coefficient, non-log scale for calculation and log-scale axis for visualization. We found a significant positive correlation between TET1 and GSN expression in human OVCA tissues (p = 0.00044), CHOL (p = 2.2e^− 33^), CESC (p = 0.0063), and READ (p = 3.7e^− 06^). (Fig. 2B).

### pGSN is highly expressed in chemoresistant OVCA cells compared to their sensitive counterpart

We validated previous findings from our lab where we reported that OVCA chemoresistant cells express high pGSN compared to chemosensitive cells; a phenomenon that is also associated with decreased chemoresponsiveness of chemoresistant OVCA cells [13]. Endometrioid (sensitive, A2780s; resistant, A2780cp) and HGS (sensitive, TOV3133G; resistant, TOV3133R) cell lines were cultured with cisplatin (cis-diamminedichloroplatinum; CDDP) (10 µM) and dimethyl sulfoxide (DMSO; 0.1%) as a control for 12–48 h. Significant increase in apoptosis (p < 0.0001) and reduction in viable cell % (p < 0.0001) were observed after CDDP treatment in chemosensitive cells compared to chemoresistant cells (Fig. 2C). qPCR and Western blot analysis showed significantly higher pGSN mRNA abundance [A2780cp (p = 0.0089), TOV3133R (p = 0.0002)] and protein content [A2780cp (p < 0.0001), TOV3133R (p = 0.0039)] in resistant cells compared to their sensitive counterparts. CDDP treatment significantly decreased pGSN protein contents (p = 0.0112) (Fig. 2D) and mRNA (p = 0.0049) (Fig. 2E) in sensitive cells (A2780s) but not in resistant cells.

### TET1 is differentially expressed between chemosensitive and chemoresistant OVCA cells

**Fig. 3:**
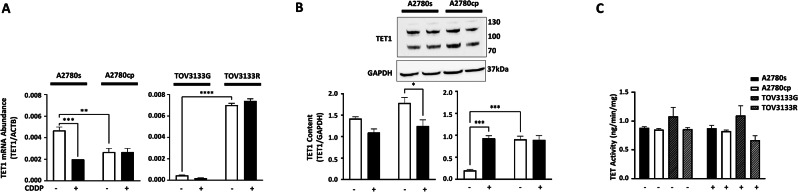
TET1 is differentially expressed between chemosensitive and chemoresistant OVCA cells, however, no significant differences in the TETs activity observed different OVCA cell types. OVCA cells were treated with or without CDDP (10 µM; 24 h). (**A**) TET1 mRNA content relative to ACTB (loading control) was assessed by qPCR, (**B**) TET1 and GAPDH (loading control) protein contents were assessed by Western blotting, and (**C**) TET activity was assessed using epigenase 5mC-hydroxylase TET activity assay kit (mean ± SEM; n = 3). Differences between all the groups were evaluated using two-way ANOVA followed by Tukey’s multiple comparison test; p*<0.05, p**<0.01, p***<0.001, p****<0.0001

In endometrioid OVCA cells, significantly higher TET1 mRNA content was observed in chemosensitive cells (A2780s) compared to resistant counterpart (A2780cp) (p = 0.0052). Additionally, CDDP (10 µM; 24 h) significantly decreased TET1 mRNA content in sensitive cells but not in resistant cells (p = 0.0008) (Fig. 3A). However, contrary to mRNA levels, high TET1 protein content was observed in chemoresistant cells compared to their chemosensitive counterparts. Western blot analysis revealed bands at two different positions (between ~ 70kDA- 130 kDa). The basal levels of both the upper and lower bands of TET1 are higher in resistant cells compared to chemosensitive cells. The upper TET1 band is decreased in both cells after CDDP treatment; however, the lower band is unaffected in the resistant but upregulated in the sensitive cells after CDDP treatment (Fig. 3B). Although different antibodies against TET1 were used to analyze its protein content, we were unsuccessful in getting bands at the expected position (~ 235 kDa). In HGS cells, however, we observed significantly higher TET1 mRNA content in resistant cells (TOV3133R) compared to sensitive counterpart (TOV3133G) (p < 0.0001). Although a slight decrease in TET1 was observed in the sensitive cells after CDDP treatment, this change was not significant (Fig. 3A). These findings suggests that TET1 expression might be histological subtype dependent in OVCA cells. Cell lysates obtained after CDDP treatment were also used to quantify the enzymatic activity of TETs by measuring the hydroxylase activity as per manufacturer’s protocol, using epigenase 5mC-hydroxylase TET activity assay kit. We did not observe any significant differences in the TETs activity among sensitive (A2780s, TOV3133G) and resistant (A2780cp, TOV3133R) OVCA cells with and without CDDP treatment (Fig. 3C).

Taken together, above findings suggests that TET1 expression in OVCA cells might be histological dependant whereas TETs enzymatic activity does not alter between sensitive and resistant cells irrespective of the presence of CDDP.

### Methylation changes induced by DNMT/TETs inhibitor alters the pGSN expression and CDDP-induced apoptosis in OVCA cells

**Fig. 4:**
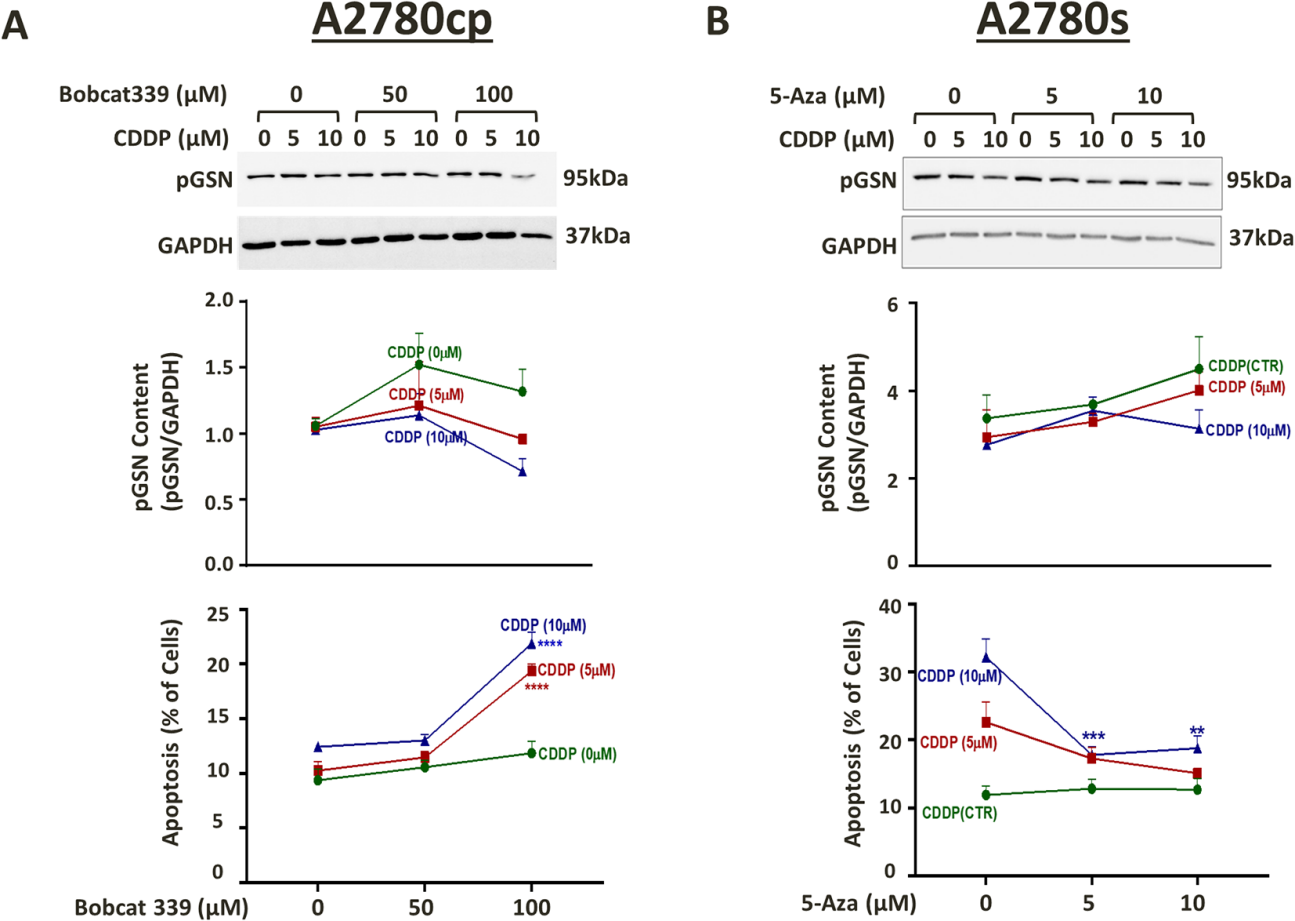
DNA methylation plays a role in the regulation of pGSN expression in OVCA cells. A2780cp and A2780s cells were treated with (**A**) BobCat 339; TETi (0-100 µM; 48 h) and (**B**) 5-aza; DNMTi (0–10 µM; 48 h), respectively, followed by CDDP treatment (10 µM; 24 h). pGSN and GAPDH (loading control) protein contents were measured by Western blotting and apoptosis was morphologically determined by Hoechst 33,258 nuclear staining (mean ± SEM; n = 3). (p*<0.05, p**<0.01, p***<0.001, p****<0.0001 versus the corresponding control group: BobCat 339/5-aza; 0 µM)

**Fig. 5:**
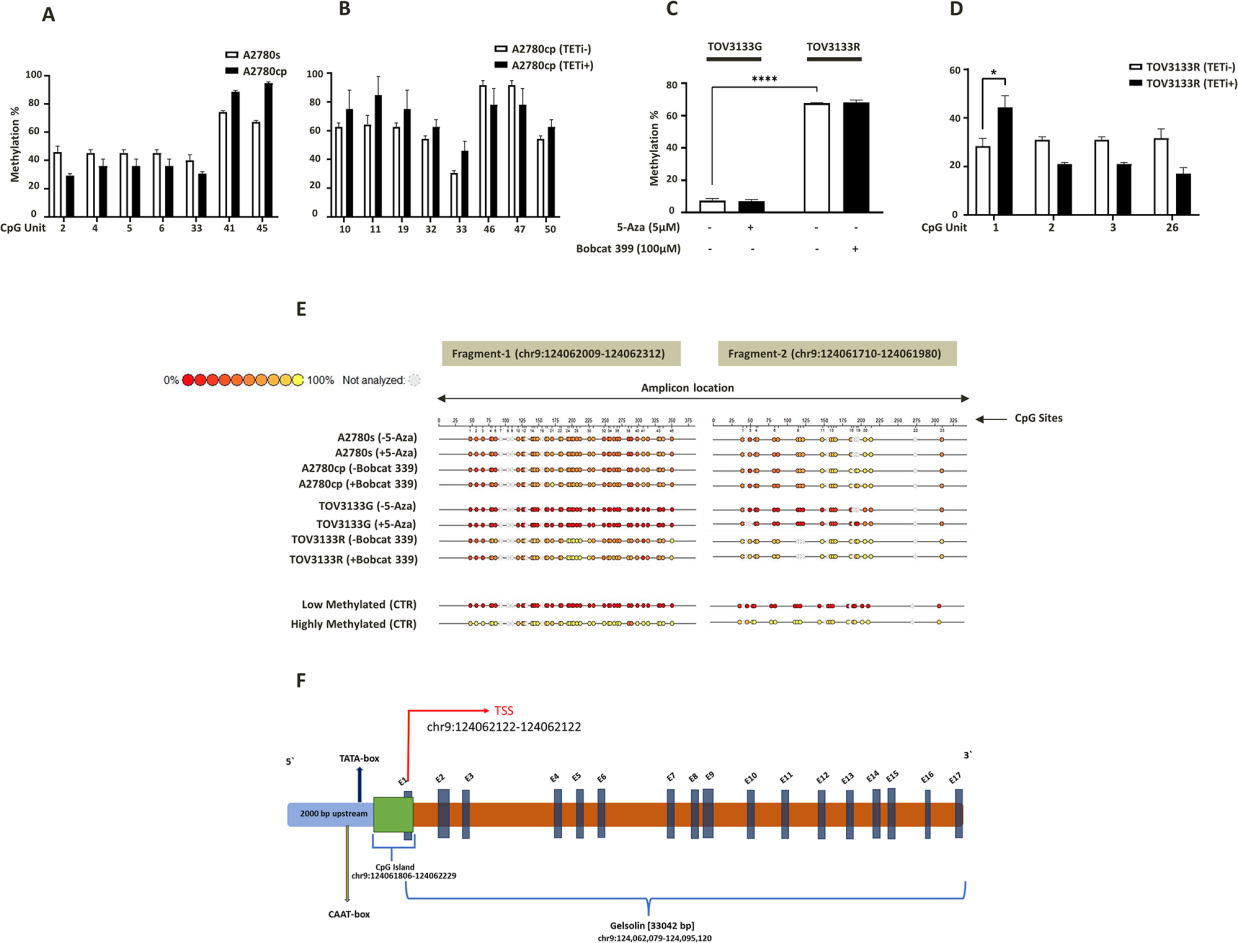
DNA methylation alters pGSN methylation in OVCA cells. Chemoresistant (A2780cp, TOV3133R) and chemosensitive (A2780s, TOV3133G) OVCA cells of were treated with BobCat 339; TETi (100 µM; 48 h) and 5-aza; DNMTi (5 µM; 48 h), respectively. DNA methylation percentage was quantitatively assessed by EpiTYPER DNA methylation technology. Methylation % differences (**A**) at multiple CpG sites between endometrioid sensitive (A2780s) vs. resistant (A2780cp) cells, (**B**) at multiple CpG sites in endometrioid resistant cells after TETi treatment (**C**) between HGS sensitive (TOV3133G) and resistant (TOV3133R) cells (**D**) at multiple CpG sites in HGS resistant cells after TETi treatment (**E**) schematic diagram of methylation status (**F**) schematic diagram of GSN gene. The region of interest for pGSN was analyzed in 2 fragments. Circles mark the position of CpG sites and color indicates the methylation status of CpG site. The color scale in each top left corner indicates the methylation level. Circle color from red to yellow indicates the methylation ranging from 0-100%. (mean ± SEM; n = 3)

To investigate the role of DNA methylation in the regulation of pGSN expression, we induced hypermethylation and hypomethylation in chemoresistant and chemosensitive cells, respectively, by pharmacological inhibition of DNMTs and TETs and investigated its effect on pGSN expression and chemoresponsiveness of OVCA cells. Chemoresistant (A2780cp) and sensitive (A2780s) OVCA cells were treated with BobCat339 – an inhibitor of TETs (TETi, 0-100 µM; 48 h) and 5-Azacytidine - an inhibitor of DNMTs (DNMTi, 0–10 µM; 48 h) respectively. Treated cells were harvested to quantify methylation ratio in pGSN promoter region (including ~ 600 bp upstream region) by EpiTYPER technology (Fig. 5). A2780cp and A2780s cells were also treated with CDDP (10 µM; 24 h) following TETi and DNMTi treatment and pGSN protein content as well as chemo-responsiveness (apoptosis %) analyzed (Fig. 4).

As demonstrated in our previous findings, we observed CDDP treatment was more effective in reducing pGSN expression and inducing apoptosis in chemosensitive cells compared to chemoresistant cells (Fig. 2C). Whereas TETi treatment alone had no significant influence on pGSN content and apoptosis in resistant cells, it markedly enhanced the CDDP-induced pGSN suppression and CDDP-induced apoptosis (Fig. 4A). In the absence of DNMTi treatment, CDDP decreased pGSN content and induced apoptosis. DNMTi treatment alone increased pGSN content in sensitive cells and markedly attenuated the CDDP-induced pGSN suppression; responses that was associated with suppressed CDDP-induced apoptosis (Fig. 4B).

In endometrioid OVCA cells, methylation analysis of pGSN promoter region including 5`-upstream region revealed hypomethylation at five and hypermethylation at two CpG dinucleotides in resistant cells (A2780cp) compared to sensitive cells (A2780s) (Fig. 5A) (Table 1). Whereas DNMTi treatment in sensitive cells did not induce any change in pGSN methylation level, TETi treatment in resistant cells appears to induce hypermethylation at five and hypomethylation at three CpG dinucleotides in pGSN promoter upstream region (Fig. 5B) (Table 1) suggesting hypomethylation role of TETs in the regulation of pGSN in OVCA cells. In the case of HGS (TOV3133R and TOV3133G), hypermethylation (average 68%) across all CpG sites at pGSN promoter region is observed in resistant cells (TOV3133R) and hypomethylation (average 8%) in sensitive cells (TOV3133G) (Fig. 5C). Similar to our observation in endometrioid cells, DNMTi treatment did not induce any changes in pGSN methylation level in sensitive cells however TETi treatment appear to induce hypermethylation at one and hypomethylation at three CpG dinucleotides in pGSN promoter upstream region (Fig. 5D) (Table 1) suggesting methylation role of TETs in the regulation of pGSN in HGS cells as well.

**Table 1:** Methylation changes observed in multiple CpG sites at pGSN promoter region including 5`-upstream region (GRCH37-hg19: chr9:124061710–124,062,312)

CpG Unit	Position	CpG Unit	Position
CpG_1	chr9:124062312	CpG_26	chr9:124062052
CpG_2	chr9:124062303	CpG_32	chr9:124061980
CpG_3	chr9:124062293	CpG_33	chr9:124061970
CpG_4	chr9:124062281	CpG_41	chr9:124061872
CpG_5	chr9:124062279	CpG_45	chr9:124061833
CpG_6	chr9:124062274	CpG_46	chr9:124061826
CpG_10	chr9:124062211	CpG_47	chr9:124061824
CpG_11	chr9:124062189	CpG_50	chr9:124061710
CpG_19	chr9:124062127		

Taken together, the above findings (Figs. 4 and 5) suggest that pGSN is regulated by DNA methylation, especially by TETs, in OVCA cells and is associated with chemo-responsiveness.

### TET1 has inverse association with pGSN expression in OVCA cells

**Fig. 6:**
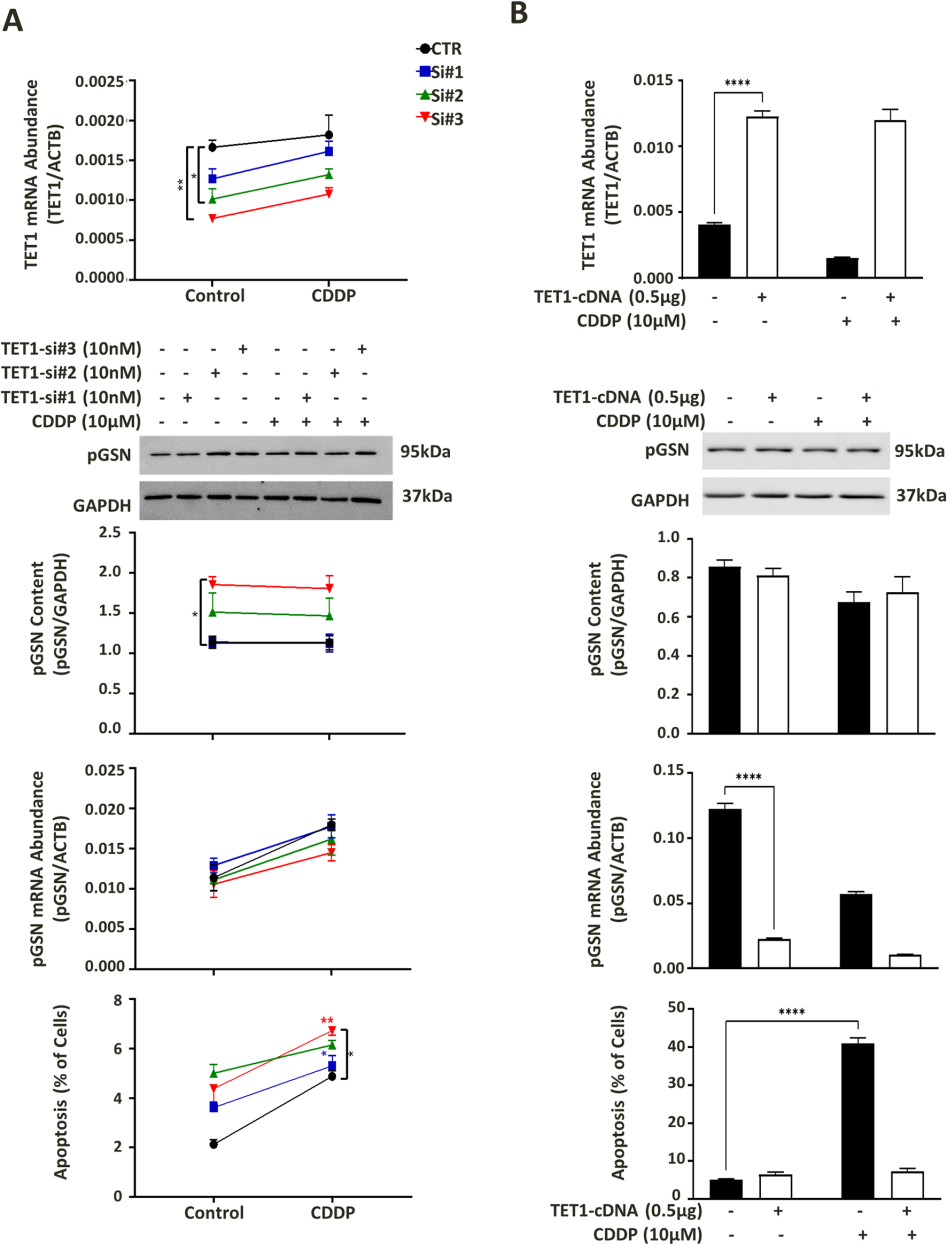
TET1 appears to have an inverse relationship with pGSN expression. (**A**) A2780cp cells were transfected with three TET1-siRNAs (10 nM; 24 h) and (**B**) A2780s cells were transfected with TET1-cDNA (0.5 µg; 24 h), both followed by CDDP treatment (10 µM; 24 h). mRNA contents relative to ACTB (loading control) were assessed by qPCR. pGSN and GAPDH (loading control) protein contents were measured by Western blotting and apoptosis morphologically determined by Hoechst 33,258 nuclear staining (mean ± SEM; n = 3). p*<0.05, p**<0.01, p***<0.001, p****<0.0001

Increasing evidence has revealed the role of TET1 in hypomethylating candidate genes that leads to cancer development and chemoresistance [34, 35, 37]. After confirming the role of DNA hypomethylation in the regulation of pGSN expression we further investigated specifically the role of TET1 in pGSN expression and pGSN-mediated chemoresistance in OVCA cells. TET1 was overexpressed and silenced in chemosensitive and chemoresistant OVCA cells respectively and its effect on the pGSN content and chemo-responsiveness were analyzed. Chemoresistant cells (A2780cp) were transfected with three different siRNAs targeting TET1 (hs. Ri. TET1.13.1, hs. Ri. TET1.13.2, hs. Ri. TET1.13.3; 10nM) and negative scramble control siRNA (DS NC1; 10 nM) with lipofectamine RNAiMax transfection reagent followed by CDDP treatment (10 µM; 24 h). Transfection efficiency was confirmed with A2780cp cells using positive control (HPRT-S1 DS positive duplex control) and fluorescently labeled transfection control (TYE™ 563 DS transfection control). TET1 knockdown significantly increased pGSN protein content and CDDP-induced apoptosis in endometrioid resistant cells; A2780cp (Fig. 6A).

In contrast, TET1 was overexpressed in chemosensitive cells (A2780s) using TET1 cDNA (TET1 cDNA ORF clone, Human, SinoBiological; 0.5 µg) and an empty vector control (pCMV3-C-GFPSpark® vector, SinoBiological) with lipofectamine 2000 transfection reagent followed by CDDP treatment (10 µM; 24 h). Overexpression of TET1 significantly reduced pGSN mRNA content in endometrioid sensitive cells (A2780s) but not pGSN protein content. Also, significant decrease in CDDP induced apoptosis was observed (Fig. 6B). Taken together loss-of-function and gain-of-function studies implied that pGSN expression in OVCA cells is inversely regulated by TET1 expression which is also associated with chemo-responsiveness.

## Discussion

Platinum-based chemotherapy with surgical debulking is the gold standard treatment approach for ovarian cancer [47], however, its clinical effectiveness is greatly influenced by the development of chemoresistance in OVCA patients. Therefore, exploration of cellular and molecular mechanism underlying chemoresistance development in OVCA patients is important for development of targeted therapies and improvement of overall survival rate. DNA methylation, both at gene-specific and global level, is arguably the most widely studied epigenetic mechanism with regards to OVCA development, progression, recurrence and chemoresistance [24, 29, 30, 48, 49]. DNA methylation is reversible and thus inhibition of DNA methylation/de-methylation can be considered as a promising therapeutic approach for OVCA treatment. Several epigenetic drugs targeting DNA methylation have been approved by FDA in different cancer cell types, including 5-azacytidine and 5-Aza-2-deoxycytidine for the treatment of myelodysplastic syndrome; MDS and adult acute myeloid leukemia; AML [50, 51].

pGSN is highly expressed in OVCA resistant cells compared to sensitive cells and its expression is associated with decreased chemoresponsiveness [13]. In this study, we demonstrated the contribution of DNA methylation, specifically TET1-induced hypomethylation, in the regulation of pGSN expression and pGSN-induced chemoresistance in OVCA cells. To support the rationale of our hypothesis, we stratified public datasets to find the correlation between pGSN and TET1 and we identified positive correlation between pGSN and TET1 in multiple human cancer. Previous studies have shown that cisplatin-resistant endometrioid OVCA cells have increased levels of TET1 compared to sensitive cells which was associated with reduced apoptosis [37]. Contrary to previous findings, we have identified a lower TET1 expression in endometrioid resistant cells compared to their sensitive counterpart. Meanwhile, TET1 was overexpressed in HGS resistant cells compared to sensitive cells. These findings suggest that TET1 is differentially expressed in different histological subtypes of OVCA, supporting the concept for its importance in personalized therapies. Further studies are however, needed to further validate histological-specific behaviour of TET1 in OVCA cells using multiple cell lines of different subtypes.

Although pGSN is transcriptionally regulated in OVCA chemoresistance, we have yet to determine how methylation affects its function. Pharmacological inhibition of DNA methylating and demethylating agents altered pGSN protein levels and chemoresponsiveness of the OVCA cells, suggesting that DNA methylation regulates pGSN expression and affect pGSN-mediated cisplatin resistance in OVCA cells. The upstream region (~ 600 bp) of pGSN promoter region is highly enriched with G/C and contains almost 69 CpG sites. We observed hypomethylation at multiple CpG sites in pGSN promoter upstream region in resistant cells compared to sensitive cells. Further, we observed increase in methylation at multiple CpG sites in pGSN promoter upstream region after inhibiting TETs which pointed to the conclusion that DNA methylation, specifically TETs induced hypomethylation appears to play a role in pGSN regulation in OVCA cells. The regulation of pGSN in ovarian cancer is complex. Although methylation changes observed after inhibitor treatment provided a valuable information about epigenetic regulation of pGSN, the association was not statistically significant. Besides directly affecting methylation at pGSN promoter, other factors may also contribute to pGSN regulation by DNA methylation. pGSN upregulates its own expression via the α5β1 integrins / FAK / Akt / HIF-1α axis [13]. It has been shown that DNA methylation can influence HIF-1α stability and subsequently affect expression of its downstream gene targets [52]. Hence, it is possible that treatment of methylating/demethylating agents resulted in DNA methylation changes in HIF-1α which subsequently modified pGSN expression.

DNA methylation is negatively correlated with chromatin accessibility and transcription factor binding sites and act as a transcriptional repression [53]. We have identified multiple transcription factor binding sites (Table 2) within GSN promoter sequence region that have previously been reported to be regulated by DNA methylation and are associated with chemoresistance in OVCA including Signal transducer and activator of transcription (STAT3) [54], Msh homeobox 1 (MSX-1) [29], cAMP response element-binding protein (CREB), octamer-binding protein (OCT-1) [55]. Treatment of DNMTi/TETi in OVCA cells might have resulted in transcriptional repression or activation of pGSN through these transcription factors. Therefore, further studies need to be conducted to confirm which upstream mechanism was altered because of induced methylation/demethylation that ultimately affected pGSN expression.

**Table 2:** Transcription factor binding sites identified within the GSN promoter sequence region (prediction and visualization using match 1.0 weight-based matrix program from TRASNFAC 6.0. (http://gene-regulation.com/pub/programs.html#match)

Name	Sequence	Position (0-based)	Strand	Score	p-value	E-value
STAT3 (M00497)	GGGTTCCC	179	-	7.35	0.00045	0.188
Msx-1 (M00394)	CACTAAATG	70	-	8.71	5.0E-5	0.0208
CREB (M00177)	CCTGACATACGG	98	-	9.05	0.000575	0.237
Oct-1 (M00162)	AGGCCTGACATACG	99	-	8.73	0.000975	0.40
YY1 (M00059)	TGCGCCCATTTAGTGTG	64	+	9.47	0.00015	0.061

STAT3, Signal transducer and activator of transcription; MSX-1, Msh homeobox 1, CREB, cAMP response element-binding protein; OCT-1, octamer-binding protein

Multiple studies have reported the involvement of TET1 in hypomethylating candidate genes that leads to cancer development and chemoresistance [34, 35, 37]. Information from OVCA public datasets showed TET1 expression in OVCA tissue is significantly associated with GSN expression (Fig. 2A). Gain- and loss-of-function assays showed TET1 is inversely related to pGSN and positively related to chemoresponsiveness of OVCA cells (Fig. 6). In our study, TET1 knockdown resulted in increased pGSN expression and increased chemoresponsiveness. This is contrary to previous studies where pGSN overexpression was significantly associated with decreased chemoresponsiveness of OVCA cells [13]. Silencing TET1 increases cisplatin sensitivity by altering the expression of multiple genes in various pathological conditions including vimentin in OVCA [37], and o6-methylguanine-DNA methyltransferase (MGMT) in oral squamous cell carcinoma (OSCC) [56]. This opposite relation observed between pGSN expression and chemoresistance after silencing TET1 could therefore be explained by the effect of other TET1 targets that suppressed the resistant effects of pGSN and enhanced cisplatin sensitivity. Although we observed a negative relation between TET1 expression and pGSN expression in OVCA cells, we did not examine the methylation regulation of TET1 on pGSN promoter region. Hence, the underlying mechanisms upregulating pGSN expression after TET1 knockdown needs to be investigated.

Although, several studies have demonstrated the importance of TET1, the mechanism through which TET1 regulates gene expression remains controversial [34, 38, 57–60]. TET1 exerts tumor suppressor function by hypomethylating tumor suppressor genes and reactivating their expression [38, 59, 60]. However, emerging evidence has revealed oncogenic role of TET1-dependent and independent of its dioxygenase activity, suggesting the existence of its non-catalytic function in regulating gene expression [34, 57, 58]. A dual role of gelsolin as tumor suppressor and oncogene has been observed in different human cancers [46]. Therefore, further studies with TET1 knockdown and overexpression are needed to determine whether the dysregulation in pGSN expression is because of pGSN promoter demethylation or transcriptional activation/repression by non-catalytic function of TET1.

Although the findings from this study are compelling, we acknowledge two main limitations. Our study was conducted using only one pair (sensitive/resistant) of OVCA cell lines from two dominant histological subtypes-HGSOC and endometrioid. Multiple cell lines from different histological subtypes, in-vivo models including animal models and patient-derived tissue need to be included in future studies to validate the role of DNA methylation, pGSN and TET1 in OVCA chemoresistance. Secondly, TET1 knockdown and overexpression conditions were optimized using endometrioid cell lines (A2780s and A2780cp). Despite being one of the most used cell line models, A2780 cell lines are not similar in their histopathological origin with HGSOC. Considering TET1 expression appears to be dependent on histology of cell lines, knockdown condition needs to be optimized in every histologic subtype cell line.

## Conclusion

In conclusion, we have demonstrated that pGSN and TET1 are differentially expressed between OVCA chemosensitive and chemoresistant cells and their expression is also associated with chemoresponsiveness of OVCA cells. Furthermore, we revealed that DNA methylation, specifically TETs induced hypomethylation appears to regulate pGSN expression in OVCA cells and TET1 has an inverse relationship with pGSN expression. DNA methylation is reversible and thus inhibition of DNA methylation/de-methylation can be considered as a promising therapeutic approach for OVCA treatment. This study provides mechanistic insights into the epigenetic regulation of pGSN relevant to chemotherapy resistance in OVCA. This regulatory mechanism of pGSN reveals a molecular basis for personalized OVCA therapy, and targeting pGSN methylation holds a great promise in overcoming OVCA chemoresistance and to improve overall OVCA patient survival. One possible biotechnological tool for this purpose could be fusion of TETs demethylases with recombinant transcription activator-like effectors (TALE) to guide the demethylases to the pGSN promoter region and modify the methylation level [61]. Another tool could be CRISPR-dCas9 mediated TET1 targeting for selective demethylation of pGSN [62]. Epigenetic drugs when used in combination with chemotherapeutic drugs has reported to be more effective than either treatment alone [63]. Combination of pGSN-specific epigenetic drugs with chemotherapy may have potential in overcoming drug resistance in OVCA.

## Materials and methods

### TCGA dataset analyses

Publicly available ovarian cancer dataset (www.kmplot.com) were interrogated to identify the association of pGSN (affymetrix Id: 200696_at) expression, and TET1 (affymetrix Id: 228904_at) expression with OVCA patients’ survival. Patient information was stratified based on histological subtype (serous), surgical outcome (optimal/suboptimal debulking), and chemotherapy treatment (platinum) [64]. Further, all tumor stages, grades and p53 statuses were included in the analysis. Progression-free survival (PFS) and overall survivals (OS) of OVCA patients were correlated with TET1 and GSN expression using Kaplan Meier plotter. The log-rank test was used for statistical parameter calculation and graph plot was used for visualization [64]. GEPIA (www.gepia.cancer-pku.cn) public dataset was used to demonstrate the correlation between pGSN and TET1 gene expression in ovarian cancer (OVCA), cholangiocarcinoma (CHOL), cervical squamous cell carcinoma and endocervical adenocarcinoma (CESC), and rectum adenocarcinoma (READ) tissues. Statistical parameters were calculated using spearman correlation coefficient, non-log scale for calculation and log-scale axis for visualization [65].

## Reagents

Cis-diaminedichloroplatinum (CDDP;cisplatin, Cat# P4394), 5-azacytidine (Cat# A2385), Bobcat339 (Cat # SML2611), anti-pGSN antibody (anti-gelsolin antibody, mouse monoclonal, Cat # SAB4200750) and dimethyl sulfoxide (DMSO, Cat # D8418) were purchased from Sigma-Aldrich (Oakville, Canada). TriFECTa® RNAi kit (hs.Ri.TET1.13) was purchased from Integrated DNA Technology (Iowa, USA) for gene silencing assays, whereas TET1 cDNA (TET1 cDNA ORF Clone, Human, C-GFPSpark® tag, Cat # HG19726-ACG) and empty control vector (pCMV3-C-GFPSpark, Cat# CV026) were purchased from SinoBiological (South Carolina, USA). Anti-TET1 antibodies were purchased from Abcam (Toronto, Canada); Cat# ab272900, ab272901, ab191698, Thermofisher scientific (Nepean, Canada); Cat# PA5-85489, and Active motif (California, USA); Cat# 91,171. Primers (TaqMan gene expression assays; TET1 (Cat # 4,331,182, Assay ID: Hs00286756_m1), pGSN (Cat # 4,331,182, Assay ID: Hs00609272_m1), and ATCB (Cat# 4333762T)), cDNA synthesis kit (High-Capacity cDNA reverse transcription kit, Cat # 4,368,814) and Hoechst 33,258 (Cat# 94,403) stain were bought from Thermofisher scientific (Nepean, Canada). Extraction kits for RNA (RNeasy mini kit, Cat# 74,104) and DNA (AllPrep DNA/RNA mini kit, Cat#80,284, DNeasy blood & tissue kit, Cat # 69,504) were from Qiagen (Toronto, Canada). TET activity kit (Epigenase 5mC-Hydroxylase TET activity/Inhibition assay kit, Cat# P-3086) was purchased from EpigenTek Group Inc (New York, USA). TET1 (Cat#31,417), TET2 (Cat#31,418), and TET3 (Cat#31,421) recombinant proteins were from Active motif, Inc, (California, USA).

## Cell lines and cell culture

Chemosensitive and chemoresistant OVCA cell lines of endometrioid and HGS histologic subtypes were used in this study. Endometrioid cell lines (chemosensitive; A2780s, chemoresistant; A2870cp) were generously donated by Dr. Barbara Vanderhyden (Ottawa Hospital Research Institute, Ottawa, ON, Canada) and HGS cell lines (chemosensitive; TOV3133G, chemoresistant; TOV3133R) were kindly provided by Dr. Anne-Marie Mes-Masson (Centre de recherche du Centre hospitalier de l’Université de Montréal (CRCHUM), Montreal, QC, Canada). The characteristics of the cells are as follows: endometrioid cells [A2780S (p53 wildtype- sensitive) A2780CP (p53 mutant; V127F, R260S-resistant)], and HGS [TOV3133G (nonsense-sensitive), TOV3133R (p53 mutant; Gln192Ter-resistant) [66–68]. All the cell lines were validated at Sick Kids Centre for Applied Genomics Genetic Analysis Facility. Endometrioid and HGS cell lines were cultured in RPMI-1640 and OSE media respectively, with 10% fetal bovine serum (FBS) at 37ºC in humidified 5% CO_2_ incubator. For cisplatin treatment, 10 µM of cisplatin was added to the cells in the absence of serum [69].

## RNA interference

Chemoresistant cells (2 × 10^5^ cells) were transfected with three different siRNAs targeting TET1 (hs.Ri. TET1.13.1,2,3; Integrated DNA Technology (IDT); 0–20 nM) and negative scrambled control siRNA (Dicer Substrate (DS) Negative Control 1; IDT) with lipofectamine RNAiMax transfection reagent for 48 h followed by CDDP treatment (0–10 µM; 24 h). Using multiple siRNA helps to rule out off target effects of gene silencing [69, 70]. Transfection efficiency was confirmed by using positive control (HPRT-S1 DS positive duplex control; IDT) and fluorescently labeled transfection control (TYE™ 563 DS transfection control: IDT). Successful knockdown was confirmed by qPCR as per TaqMan gene expression assay protocol.

## Transient transfection

Chemosensitive cells (2 × 10^5^ cells) were transiently transfected with TET1 cDNA (TET1 cDNA ORF clone, Human, SinoBiological; 0–2 µg; 24 h) and an empty vector control (pCMV3-C-GFPSpark® vector, SinoBiological; 0–2 µg; 24 h) using lipofectamine 2000 transfection reagent, followed by CDDP treatment (0–10 µM; 24 h) [69, 71, 72]. Successful knockdown was confirmed by qPCR as per TaqMan gene expression assay protocol.

## Pharmacological inhibition of DNMTs and TETs

Chemosensitive and chemoresistant OVCA cells (1 × 10^6^ cells) were treated with 0–10 µM 5-Azacytidine (DNMTi) and TETs inhibitors (BobCat339; 0-100 µM), respectively, once a day for two days followed by 0–10 µM cisplatin (24 h). After cisplatin treatment, cells were harvested for subsequent analysis.

## RNA extraction and quantification

Total RNA was extracted from cells (1 × 10^6^) using RNeasy mini kit (Qiagen) according to the manufacturer’s protocol. RNA concentration and purity were confirmed using Nanodrop spectrophotometer. cDNA was synthesized using High-Capacity cDNA Reverse Transcription Kit (Thermofisher scientific) according to manufacturer’s protocol. Quantitative polymerase chain reaction (qPCR) was performed using TaqMan gene expression assay system on Applied Biosystems 7500 Fast Real-Time PCR System. mRNA levels of target genes were quantified relative to ACTB mRNA level. All reactions and experiments were carried in triplicates.

## Protein extraction and quantification

Cells were collected after trypsin treatment, washed with phosphate-buffered saline (PBS), centrifuged (13,000 g for 5 min) and suspended in a complete lysis buffer (cOmplete, Mini protease inhibitor cocktail tablets dissolved in cOmplete lysis-M reagent-Roche). Samples were sonicated (30% amplitude for 30 s), and the cell lysate collected after centrifugation for 30 min (13,000 RPM at 4ºC). Protein concentration was measured using DC (Detergent compatible) protein assay (Bio-Rad). Equal amounts of protein were loaded into 10% SDS-PAGE gel for electrophoresis and transferred to nitrocellulose membrane. After protein transfer, membranes were blocked with 5% blotto (skim milk in TBST buffer; 1 h) followed by overnight incubation at 4ºC with primary antibodies (1:1000) and ultimately incubation with respective horseradish peroxidase (HRP)-conjugated secondary antibody (1:2000; 1 h; RT) [12, 69, 71]. Both primary and secondary antibodies were prepared in 5% blotto. After secondary antibody incubation, membranes were washed with TBST (3x; 15 min each). The protein bands were visualized using Enhanced chemiluminescent (ECL) kit (Thermofisher scientific) on ChemiDoc Imaging system (BioRad) and analyzed by measuring the densities of protein bands using Image J software.

## DNA extraction and quantification

Genomic DNA was extracted from cells (1 × 10^6^) using DNeasy Blood & Tissue Kit (Qiagen) according to the manufacturer’s protocol. DNA concentration and purity were quantified using Nanodrop spectrophotometer.

## Quantitative DNA methylation analysis

Extracted DNA from OVCA cell lines was bisulfite treated and amplified with PCR followed by purification of PCR products with shrimp alkaline phosphatase (SAP) treatment. Th genomic region of interest for pGSN spanned 602 base pairs (GRCH37-hg19: chr9:124061710–124,062,312, CpG units:69) and included coverage over the reported CpG island as well as CpG sites in the promoter region. A single-stranded RNA copy of the region of interest was generated by *in-vitro* transcription using reverse pGSN PCR primers tagged with the T7 recognition sequence. The resulting transcript was base-specifically (U-specific) cleaved followed by MALDI-TOF mass spectrometry quantification of methylation ratio and results were analyzed with EpiTYPER software [73]. The region of interest for pGSN was analyzed in 2 fragments spanning approximately 300 base pairs each. Highly methylated and low methylated human control DNA were also included in the assessment. Methylation data was screened for unreliable methylation ratios having low/high mass outside the detection limit of analyzer and more than one silent peak.

## TET enzymatic activity assay

Enzymatic activity of TETs was quantified using Epigenase 5mC-Hydroxylase TET Activity/Inhibition Assay Kit (Colorimetric) as per manufacturer’s protocol.

## Drug sensitivity assay

Cells (1 × 10^4^) were seeded in 96-well plate and treated with 10 µM CDDP for 24 h. The cell viability was assessed using cell counting kit-8 (CCK8) with an optical density of 450 nm and plotted as viable cell percentage.

## Apoptosis analysis

Morphological assessment of CDDP-induced apoptosis was performed using Hoechst 33,258 nuclear stain and visualized with fluorescence microscopy (ZOE fluorescent cell imager-BioRad). A minimum of 400–500 cells with apoptotic morphology were counted from random fields using blinded approach to avoid experimental bias. Apoptotic cells were expressed as the percentage of total cells.

### Statistical analysis

All the experiments were performed in three independent replicates and results were presented as mean ± SEM. Two-way or three-way ANOVA were performed for statistical purposes and Tukey *post-hoc* was used for multiple comparisons. Differences were considered significant at p < 0.05.

## Data Availability

Relevant data supporting the findings of this study is available within the article.
